# Repetitive peripheral magnetic stimulation alone or in combination with repetitive transcranial magnetic stimulation in poststroke rehabilitation: a systematic review and meta-analysis

**DOI:** 10.1186/s12984-024-01486-8

**Published:** 2024-10-16

**Authors:** Yong Wang, Kenneth N. K. Fong, Youxin Sui, Zhongfei Bai, Jack Jiaqi Zhang

**Affiliations:** 1 Department of Rehabilitation Sciences, The Hong Kong Polytechnic University, Hong Kong SAR, China; 2 Department of Rehabilitation, Henan Provincial People’s Hospital, Zhengzhou, Henan, China; 3 Research Centre for Assistive Technology, The Hong Kong Polytechnic University, Hong Kong SAR, China; 4 Department of Neurology and Neurorehabilitation, Shanghai YangZhi Rehabilitation Hospital, School of Medicine, Tongji University, Shanghai, China

**Keywords:** Stroke, Upper extremity, Peripheral magnetic stimulation, Transcranial magnetic stimulation, Cortical excitability

## Abstract

**Objective:**

This study aimed to comprehensively review the effects of repetitive peripheral magnetic stimulation (rPMS) alone or in combination with repetitive transcranial magnetic stimulation (rTMS) on improving upper limb motor functions and activities of daily living (ADL) in patients with stroke, and to explore possible efficacy-related modulators.

**Methods:**

A literature search from 1st January 2004 to 1st June 2024 was performed to identified studies that investigated the effects of rPMS on upper limb motor functions and ADL in poststroke patients.

**Results:**

Seventeen studies were included. Compared with the control, both rPMS alone or rPMS in combination with rTMS significantly improved upper limb motor function (rPMS: Hedge’s g = 0.703, *p* = 0.015; rPMS + rTMS: Hedge’s g = 0.892, *p* < 0.001) and ADL (rPMS: Hedge’s g = 0.923, *p* = 0.013; rPMS + rTMS: Hedge’s g = 0.923, *p* < 0.001). However, rPMS combined with rTMS was not superior to rTMS alone on improving poststroke upper limb motor function and ADL (Hedge’s g = 0.273, *p* = 0.123). Meta-regression revealed that the total pulses (*p* = 0.003) and the number of pulses per session of rPMS (*p* < 0.001) correlated with the effect sizes of ADL.

**Conclusions:**

Using rPMS alone or in combination with rTMS appears to effectively improve upper extremity functional recovery and activity independence in patients after stroke. However, a simple combination of these two interventions may not produce additive benefits than the use of rTMS alone. Optimization of rPMS protocols, such as applying appropriate dosage, may lead to a more favourable recovery outcome in poststroke rehabilitation.

**Supplementary Information:**

The online version contains supplementary material available at 10.1186/s12984-024-01486-8.

## Introduction

Repetitive peripheral magnetic stimulation (rPMS) is a non-invasive therapeutic approach for facilitating motor recovery following neurological diseases, which was first proposed for the purpose of neurological rehabilitation in 1996 [1]. The rPMS technique employs focused magnetic pulses over various peripheral targets (e.g., muscles, nerves, or spinal roots) [2], and this technique induces repetitive contraction-relaxation cycles by depolarizing neurons [3] and then provides proprioceptive inputs to afferent fibers [4–7], therefore modulating sensorimotor plasticity. In the literature, rPMS is considered a unique, promising neuromodulation technique due to its advantage of providing more deeply penetrating, focused, painless stimulation than conventional electrical stimulation provides [5, 8, 9].

In 2023, rPMS was delivered using a transcranial magnetic stimulator, which was originally used for repetitive transcranial magnetic stimulation (rTMS), and has been approved by the US Food and Drug Administration for relieving chronic pain [10]. In poststroke rehabilitation, rPMS is different from rTMS in the neural mechanism - rTMS has been extensively used to facilitate motor recovery by modulating cortical plasticity in a top-down approach [11] whereas rPMS is adopting a bottom-up approach through recruitment of proprioceptive afferents thus up-regulate the excitability of the sensorimotor areas via the ascending pathway [2, 6]. Therefore, combining central and peripheral magnetic stimulation may produce a synergistic effect on the facilitation of motor recovery after stroke [12].

The effects of rPMS for motor function of the hemiplegic upper extremity or ADL after stroke have been reviewed in previous systematic reviews, which generally have reported positive effects of rPMS [2, 8, 13–18]. However, these reviews are not free from methodological limitations. Firstly, a few reviews did not perform meta-analysis to quantitively evaluate the treatment effects [2, 14, 18]. Secondly, in the previous meta-analytic reviews, no detailed subgroup analysis or meta-regression was performed to identify the influence of different stimulation protocols, patient demographics, or patients’ clinical profiles on the treatment effect sizes [8, 13, 15, 16]. Thirdly, some reviews covered a wide range of neurological disease conditions, so the specific effect of rPMS in stroke rehabilitation was still not conclusive [2, 17]. Lastly, these reviews did not systematically investigate the effect of rPMS alone or in combination with rTMS to elaborate the possible synergistic effect of the combined interventions [2, 8, 13–18].

Therefore, a comprehensive understanding of clinical effectiveness as well as neural mechanisms underlying the therapeutic benefits of using rPMS alone or in combination with rTMS in poststroke rehabilitation is needed. Here, our review aimed to: (1) investigate the effects of these two interventional methods (using rPMS alone or in combination with rTMS) on upper limb motor function and ADL in poststroke patients, using meta-analysis; (2) identify any significant relationship between various rPMS parameters, patient demographics, clinical characteristics, and effect sizes using subgroup analyses and meta-regression; and (3) clarify the mechanisms underlying the therapeutic effects of rPMS by qualitatively assessing rPMS studies using neuroimaging and/or neurophysiological outcomes.

## Methods

This study was reported following the Preferred Reporting Items for Systematic Reviews and Meta-Analyses statements (PRISMA) [19]. This review has been prospectively registered in PROSPERO (ID: CRD42024547676).

### Search strategy

The literature search was conducted from 1st January 2004 to 6th February 2024, using databases including PubMed, MEDLINE, Web of Science, and EMBASE. The search was based on the Title/Abstract using the following keywords: “stroke” AND “upper limb” AND “peripheral magnetic stimulation.” A logical combination of keywords can be found in Table S1. Medical Subject Heading Terms were applied when searching PubMed. Two reviewers (YW and YS) independently scanned all titles, read the abstracts, and identified relevant studies. A manual screening was also conducted to identify target articles in the reference lists of previous systematic reviews. Before submitting our manuscript, an updated search from 7th February to 1st June 2024 on PubMed was additionally performed to identify newly published articles.

### Selection criteria and data extraction

Studies were included in this review if they satisfied all of the criteria listed below. Population: (P) Studies that recruited adult participants diagnosed with stroke. Intervention (I): Interventions that used rPMS applied to muscles and/or peripheral nerves of upper limbs, or cervical spinal nerves, or in combination with rTMS applied to the primary motor cortex (M1) cortical representations of the proximal or distal upper extremity; rPMS was delivered using a magnetic stimulator, including a transcranial magnetic stimulator, e.g., MagVenture and MagStim, or other magnetic stimulation devices, e.g., PathleaderTM. Comparison (C): Control with sham or no stimulation. Outcomes (O): Studies that provided at least one outcome assessing upper limb motor function or ADL (for motor functions of the hemiplegic upper limb, the Fugl-Meyer Assessment-Upper Extremity (FMA-UE) was selected as the primary outcome in our meta-analysis [20]). For measuring ADL, the modified Barthel Index (MBI) was considered; if MBI data were not available, the Barthel Index (BI) or the Functional Independence Measure (FIM) were used because both instruments are similar to the MBI [21, 22]. Study design (S): Randomized or nonrandomized controlled trials were included in quantitative analysis, while studies without control groups were qualitatively described.

Studies meeting any of the following criteria were excluded: (1) the study only enrolled participants with other neurological disorders excluding stroke; (2) the study was a case study with a single participant; (3) the study was published as conference abstracts, dissertations, or in books; or (4) the study was not published in English language.

To elaborate the effect of using rPMS alone or in combination with rTMS, we performed the following three subgroup meta-analyses:

#### Group A

rPMS alone versus sham or no stimulation.

#### Group B

rPMS combined with rTMS versus sham or no stimulation.

#### Group C

rPMS combined with rTMS versus rTMS alone.

### Data extraction and quality assessment

Relevant data and methodological quality of the included articles were extracted and assessed by two authors independently (YW and YS). Furthermore, we used the Physiotherapy Evidence Database (PEDro) rating scale to appraise the methodological quality of controlled trials included in the meta-analysis [23]. Any discrepancy was resolved through discussion with a third reviewer (JZ).

### Data analysis

Quantitative analysis was performed using the Comprehensive Meta-Analysis, version 3.0. In absence of meta-analyzable data (i.e., mean and standard deviation), we first contacted the corresponding authors via email to obtain raw data. In the case of non-responsive authors, we transferred the reported data (such as median/interquartile ranges) to mean and SD, using previously validated methods [24, 25]. The change scores of outcomes (post minus pre) were included in the calculation of effect sizes in the form of Hedges’ g, which corrected the possible bias of the small sample sizes [26]. The Higgins I² statistic was used to evaluate the level of heterogeneity between studies [27]. A random-effects model was used for all meta-analyses [28].

Meta-regression analysis was performed to explore any associations between characteristics at study level (e.g., time since stroke, baseline function level, type of stroke, demographics) or rPMS parameters (e.g., frequency, total number of delivered pulses, number of pulses per session, doses per stimulation site, number of training sessions) and effect sizes of upper limb motor function and ADL [29]. Additionally, possible publication bias was statistically examined using the Egger’s test [30]. The level of significance was set at two-tailed *p* < 0.05 for all statistical analyses, except that *p* < 0.10 in the Egger’s test [31].

## Results

### Study search results

**Fig. 1:**
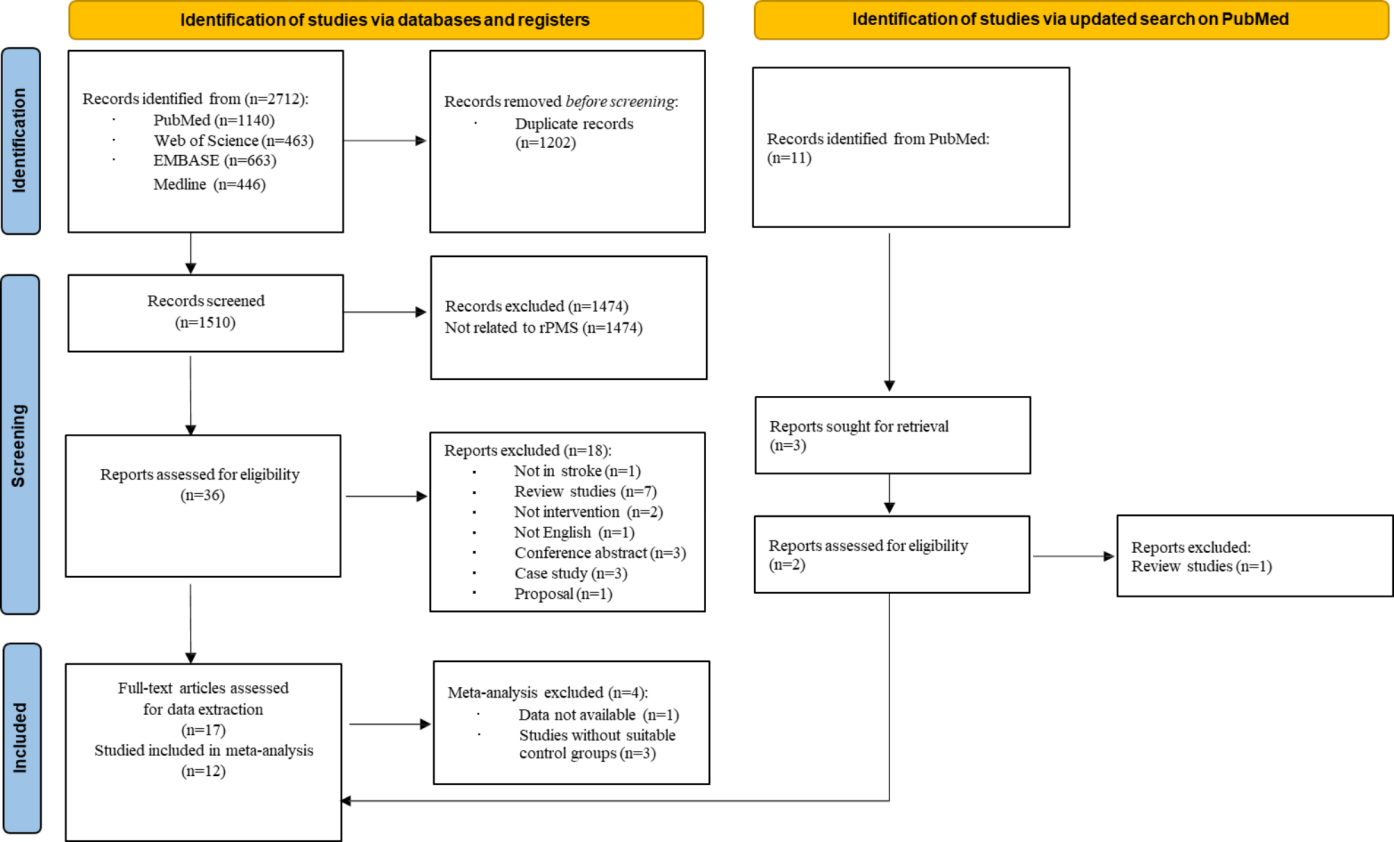
Flowchart of literature search

A total of 17 studies involving 657 participants were included in our systematic review. The process of study selection was shown in Fig. 1. Finally, we included five trials with 186 subjects which focused on the combined effects of rPMS and rTMS [12, 32–35] and 12 studies with 471 subjects investigating the effect of rPMS alone [36–47], 12 of which were included in our meta-analysis [12, 32–34, 36–38, 40, 43–45, 47]. The characteristics of the included studies are presented in Table 1.

**Table 1:** Characteristics of studies investigating the effects of rPMS alone or in combination with rTMS

		Population				rPMS protocol						Motor outcomes		
Study	Design	Group size	Chronicity	Severity of hemiplegia	Type of stroke	Protocol	Intensity	Duration	Stimulation targets	Control	Combined intervention	Clinical	Neural	Assessment timepoints
Struppler et al., 2009	Single-group	rPMS (*n* = 52)	Subacute-chronic (2 weeks–10 years)	Unclear	37 ischemic and 10 hemorrhagic strokes, 5 traumatic brain injury	rPMS-4500 20 Hz Figure-of-eight coil	Induced smooth movement of finger extension	15 min, 1 session	Finger extensor of the paretic upper limb	No	No	MAS	No	Baseline, post. follow-up at 2, 4, 24, 48, and 72 h
Krewer et al., 2014	Parallel	rPMS (*n* = 31) Sham (*n* = 32)	Subacute to chronic (≥ 2 weeks)	Unclear	60 stroke and 3 traumatic brain injury	rPMS-5000 25 Hz Butterfly coil	10% above the level that evoked a wrist or elbow movement taken at rest	2 times/day, 5 days/week, 2 weeks, 20 sessions	Flexors and extensors of the paretic wrist and elbow	Sham coil	Upper limb occupational therapy	FMA-UE MTS	No	Baseline, post, 2 weeks post
Yang et al., 2018	Parallel	rPMS (*n* = 15) Control (*n* = 15)	Acute to subacute (≤ 1 month)	Mild to moderate (muscle strength ≤ 3)	19 ischemic and 11 hemorrhagic strokes	Unclear dose 5HZ Figure-of-eight coil	100% RMT	Once a day, 5 days/week, 4 weeks, 20 sessions	Paretic supraspinatus and deltoids	Electrode stimulation	Conventional rehabilitation	FMA-UE US	No	Baseline, post
Obayashi et al., 2020	Parallel	rPMS (*n* = 10) Control (*n* = 9)	Acute (0–14 days)	Severe	16 ischemic and 3 hemorrhagic strokes	3 × 10 consecutive stimulations per muscle 30HZ Circular coil	70% MSO	Once every other day, 3 days per week, 3–32 sessions	Paretic extensor digitorum communis, extensor carpi radialis, flexor digitorum superficialis, triceps brachii, biceps brachii, and anterior or middle head of deltoid	Conventional physiotherapy	Conventional physiotherapy	WMFT FMA-UE BBT	No	Baseline, post
Fujimura et al., 2020	Single-group	rPMS (*n* = 12)	Subacute to chronic (≥ 1 month)	Mild to severe (FMA-UE: 2–33/66)	5 ischemic and 7 hemorrhagic strokes	rPMS-6000 per muscle 30HZ	Any further increase would become uncomfortable	Once a day, 5 days/week, 4 weeks, 20 sessions	Paretic supraspinatus, posterior deltoid/infraspinatus muscles	No	Conventional rehabilitation	FMA ROM NRS AHI	No	Baseline, post
Chen X et al., 2020	Parallel	rPMS (*n* = 16) LF-rTMS (*n* = 19)	Subacute	Unclear	35 ischemic strokes	Unclear doses of rPMS 30HZ Parabolic coil	20 -40% of MSO	10 sessions, 2 weeks	Extensor muscles and shoulder muscles of the paretic upper extremity	LF-rTMS	Upper limb occupational therapy	BI FMA-UE	No	Baseline, mid, post, 1 month post, 3 months post
Chen S et al., 2020	Parallel	rPMS (*n* = 16) Sham (*n* = 16)	Subacute to chronic (≥ 2 weeks)	Unclear function level	10 ischemic and 22 hemorrhagic strokes	rPMS-750, 5 Hz (MAS ≥ 1+) rPMS-5100, 20 Hz (MAS<1+) Parabolic coil	Muscle contraction threshold	1 session	Paretic shoulder adductors, extensors, elbow and wrist extensors and flexors	Sham coil	No	FMA-UE MAS MTS	EEG	Baseline, 1 session post, 24 h post
Nahas et al., 2022	Parallel	piTBS (*n* = 25) Sham (*n* = 11)	Chronic (≥ 6 months)	Unclear	Unclear	piTBS-600 50 Hz-iTBS Figure-of-eight coil	Supra-threshold intensity inducing visible muscle contraction	Once every other day, 8 sessions	Belly of paretic biceps brachii, wrist and finger flexor	Sham coil	Physical therapy	MAS	No	Baseline, post
Jiang et al., 2022	Parallel	rPMS (*n* = 24) Control (*n* = 20)	Acute-subacute (1–4 weeks)	Severe	33 ischemic and 12 hemorrhagic strokes	rPMS-2400 20HZ Circular coil	15–30% MSO inducing 30° elbow/45° wrist extension	Once a day, 2 weeks, 14 consecutive sessions	Belly of the paretic triceps brachii and extensor digitorum muscles	Conventional physiotherapy	Conventional physiotherapy	FMA-UE MBI	No	Baseline, post
Fawaz et al., 2023	Parallel	rPMS (*n* = 40) Sham (*n* = 40)	Subacute to chronic (≥ 6 weeks)	Mild to severe Shoulder abductors muscle power > grade 2)	No information	rPMS-4500 per muscle 30 Hz Circular/butterfly coil	Average 35 and 45% above the level that evoked wrist movement taken at rest	5 sessions/ week, 3 weeks, 15 sessions	Paretic shoulder abductors, elbow and wrist extensors and supinator muscle	Sham coil	Intensive upper limb occupational therapy	FMA-UE FIM ROM US	No	Baseline, post
Ke et al., 2023	Parallel	HF-rPMS (*n* = 13) Sham (*n* = 13)	Acute-subacute (4.5–42.5 days)	Severe (FMA-UE: 4-25.5/66)	26 hemorrhage strokes, 22 basal ganglia/ 4 thalami involved	rPMS-1800, 20 Hz Figure-of-eight coil	40–60% of MSO inducing significant movement of the paretic upper limb	Once a day, 10 consecutive sessions	Centre of axilla (stimulating the brachial plexus) and the popliteal fossa of paretic side (synchrous)	Sham coil	Conventional rehabilitation	FMA-UE MRC	No	Baseline, post
Fujimura et al., 2024	Parallel	rPMS (*n* = 22) Control (*n* = 24)	Acute-subacute (34 ± 23 (median = 31)) days)	Severe	23 ischemic and 23 hemorrhagic strokes	rPMS-6000 each muscle 30 Hz Circular coil	Maximum intensity without inducing pain or discomfort (0.65 ~ 0.9T)	Once a day, 6 weeks, 42 consecutive sessions	Paretic supraspinatus, posterior deltoid/infraspinatus muscles	Conventional rehabilitation	Conventional rehabilitation	FMA-UE ROM AHI NRS MAS	No	Baseline, post, 6-week follow up
Qin et al., 2023	Parallel	rPMS + LF-rTMS (*n* = 20) LF-rTMS (*n* = 15) Control (*n* = 14)	Subacute (1–6 months)	Mild to moderate (Brunnstrom stages 3–5)	49 ischemic strokes	rPMS-1200 10 Hz Figure-of-eight coil Delivered immediately after LF-rTMS.	Minimum intensity inducing subtle visible muscle contractions	Once a day, 5 days/week, 8 weeks; 40 sessions	Erb’s point of the paretic upper limb	LF-rTMS Conventional rehabilitation	Conventional rehabilitation	FMA-UE MBI MAS	fMRI	Baseline, post
Wu et al., 2023	Parallel	rPMS + HF-rTMS (*n* = 15) HF-rTMS + sham rPMS (*n* = 15) rPMS + sham HF- rTMS (*n* = 15) Sham (*n* = 15)	Subacute (2 weeks–6 months)	Moderate to severe (Brunnstrom stages I–III)	27 ischemic and 33 hemorrhagic strokes	rPMS-1000 10HZ Circular coil Delivered after rTMS	The lowest stimulation intensity that can trigger muscle contraction	Once a day, 5 days/week 3 weeks 15 sessions	Paretic C5-T1 nerve root	HF-rTMS rPMS Sham coil	Conventional rehabilitation	FMA-UE WMFT MBI Brunnstrom stage	No	Baseline, post, 3-month follow up
Yang et al., 2023	Case-series	rPMS + HF-rTMS (*n* = 4)	Chronic (7–12 months)	Unclear	4 hemorrhagic strokes Frontal and temporal lobe (*n* = 1) and basal ganglia (*n* = 3)	rPMS-750, 5 Hz (MAS ≥ 1+) rPMS-5100, 20 Hz (MAS<1+) Figure-of-eight Unclear performing order	100% RMT	Once a day, 15 days, 15 sessions	Flexor and extensor of the paretic elbow and wrist	No	Traditional rehabilitation	MAS NRS Grip, pinch strength	No	Baseline, post
Liang et al., 2024	Pilot	rPMS + HF-rTMS (*n* = 15) HF-rTMS (*n* = 15) Sham (*n* = 15)	Subacute (< 3 months)	Unclear	21 infarction and 24 hemorrhagic strokes	rPMS-1200 5 Hz Double-ended circular coil, delivered 20 ms after rTMS (paired)	80% RMT	Once a day, 5 days/week 4 weeks, 20 sessions	Paretic seventh cervical nerve root	rTMS Sham coil	Physiotherapy	FMA-UE FCA BI	TMS (MEP, RMT, SICI)	Baseline, post
Change et al., 2024	Parallel	rPMS + iTBS (*n* = 14) Sham rPMS + iTBS (*n* = 14)	Subacute to chronic (≥ 7 days)	Mild to severe FMA-UE(28.6 ± 21.3, 33.4 ± 19.7)	10 infarction and 18 hemorrhagic strokes	piTBS-600 5 Hz Figure-of-eight coil Delivered 10 min before central iTBS	Intensity inducing muscle contraction of extensor carpi radialis muscle	Once a day, 5 days/week, 2 weeks 10 sessions	Radial nerve of the paretic upper limb (radial groove)	Sham coil (low-intensity level at 5% of the MSO)	Cmprehensive rehabilitation	FMA-UE ARAT FIM-Selfcare SIS	No	Baseline, post

Abbreviations: RMT: Resting motor threshold; MSO: Maximum stimulator output; HF/LF: High/Low Frequency; FMA-UE: Fugl-Meyer Assessment Upper Extremity Score; BI: Barthel Index; MBI: Modified Barthel Index; MAS: Modified Ashworth scale; MTS: Modified Tardieu scale; ARAT: Action research arm rest; BBT: Block and Box Test; MRC: Medical Research Council scale; WMFT: Wolf motor function test; ROM: Range of motion; US: Ultrasound; NRS: Numerical Rating Scale; FIM: Functional Independence Measure; FCA: Comprehensive Functional Assessment; SIS: Stroke Impact Scale; EEG: Electroencephalography; fMRI: Functional magnetic resonance imaging; AHI: Acromion-humerus interval; piTBS: Peripheral intermittent theta burst stimulation; TMS: Transcranial magnetic stimulation; MEP: Motor evoked potential; SICI: Short interval intracortical inhibition

### Methodology quality assessment

The rating score on the PEDro scale ranged from 5 to 10, with a mean score of 8.08, which indicated that the included studies had moderate to high methodological quality (Table S2). In addition, after evaluating the funding sources for the studies included in the review, we found no evidence that funding agencies influenced the interpretation of results.

### Stimulation protocols

#### rPMS-alone stimulation protocols

In these studies, the number of rPMS pulses per stimulation target per training session ranged from 600 to 6000. High-frequency (≥ 5 Hz) rPMS protocols were used in all included studies, ranging from 5 Hz [43, 44], 10 Hz, 20 Hz [37, 38, 43, 46], 25 Hz [45], 30 Hz [36, 40–42, 47] and theta burst (50 Hz bursts repeated at 5 Hz) [39]. Regarding intensity, a supra-threshold intensity (which can evoke visible muscle contraction or significant distal movement) was most frequently used [36–40, 42–46], while some studies applied increasing intensity until any further increase induced pain or discomfort [41, 47].

#### Combined stimulation protocols

Five studies investigated the combined stimulation protocols (Table S3), with four of these studies using high-frequency rTMS [32–35] and the fifth using low-frequency rTMS [12]. Among the four studies using high-frequency rTMS, three applied excitatory stimulation to the ipsilesional hemisphere [32–34], while one applied it to the contralesional hemisphere due to participants having undergone contralateral seventh cervical nerve transfer surgery [35].

High-frequency (≥ 5 Hz) rPMS was applied in these five studies [12, 32–35]. Four studies applied rPMS and rTMS sequentially [12, 33–35], while one study synchronized the rPMS and rTMS in a paired, associative-stimulation manner [32]. In terms of rPMS intensity, when used in conjunction with rTMS, a muscle-contraction threshold was commonly applied [12, 33–35], while one study used a lower stimulation intensity with reference to the resting motor threshold (RMT) measured by TMS (i.e., 80% RMT [32]).

#### Upper extremity motor impairment

**Fig. 2:**
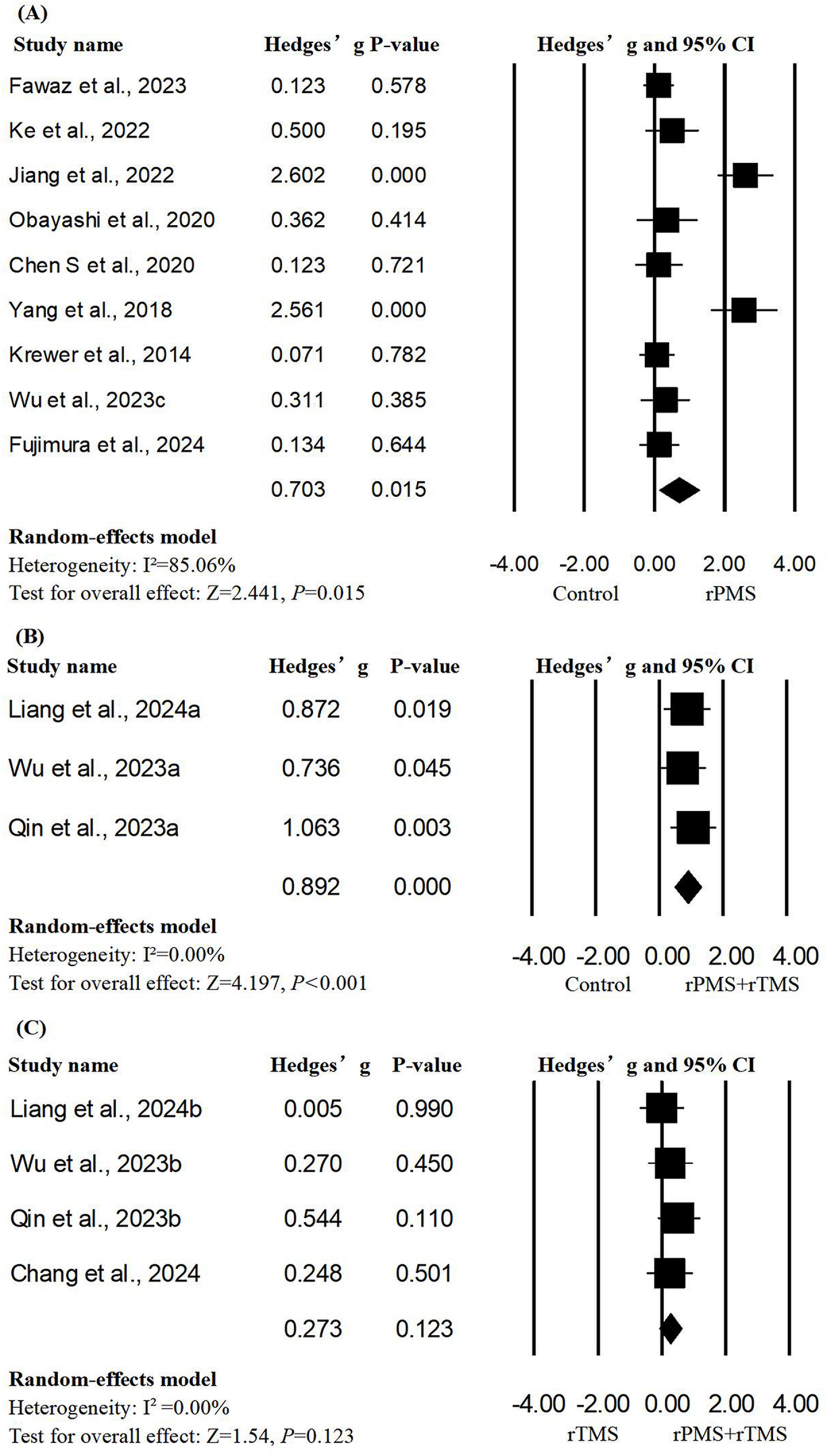
Forest plots of the pooled outcome (FMA-UE) of upper extremity motor impairment. Group **A**: Hedges’ g = 0.703, *p* = 0.015; I²=85.06%; Egger’s test: *p* = 0.05; Group **B**: Hedges’ g = 0.892, *p* < 0.001; I²=0.00%; Egger’s test: *p* = 0.64; Group **C**: Hedges’ g = 0.273, *p* = 0.123; I²=0.00%; Egger’s test: *p* = 0.40)

A total of 12 studies with 16 units of analysis were included in the meta-analysis of FMA-UE scores [12, 32–34, 36–38, 40, 43–45, 47]. When compared to the control group, the results of the meta-analysis (as shown in Fig. 1) showed that significant therapeutic effects were found in interventions both with rPMS alone and with rPMS in combination with rTMS (rPMS alone: Hedges’ g = 0.703, *p* = 0.015, I²=85.06; rPMS + rTMS: Hedges’ g = 0.892, *p* < 0.001, I²=0.00; Fig. 2), and the overall significance in each group was robust to leave-one-out sensitivity analysis (rPMS alone: Hedges’ g from 0.139 to 1.267; rPMS + rTMS: Hedges’ g from 0.475 to 1.308). Additionally, regarding the promotion of upper limb function recovery, there was not a statistically significant difference after combined stimulation protocols were compared to the use of rTMS alone (Hedges’ g = 0.273, *p* = 0.123, I²=0.00). No significant publication bias was observed according to the result of Egger’s test (rPMS + rTMS vs. control: *p* = 0.64; rPMS + rTMS vs. rTMS: *p* = 0.40), except for the rPMS-alone group (*p* = 0.05) (Figures S1–S3).

Due to the limited number of articles, we only performed meta-regression on the rPMS subgroup. Using univariate meta-regression, we failed to find any significant predictors regarding the effect size of rPMS intervention on upper limb motor function (Table S4).

### Activities of daily living

**Fig. 3:**
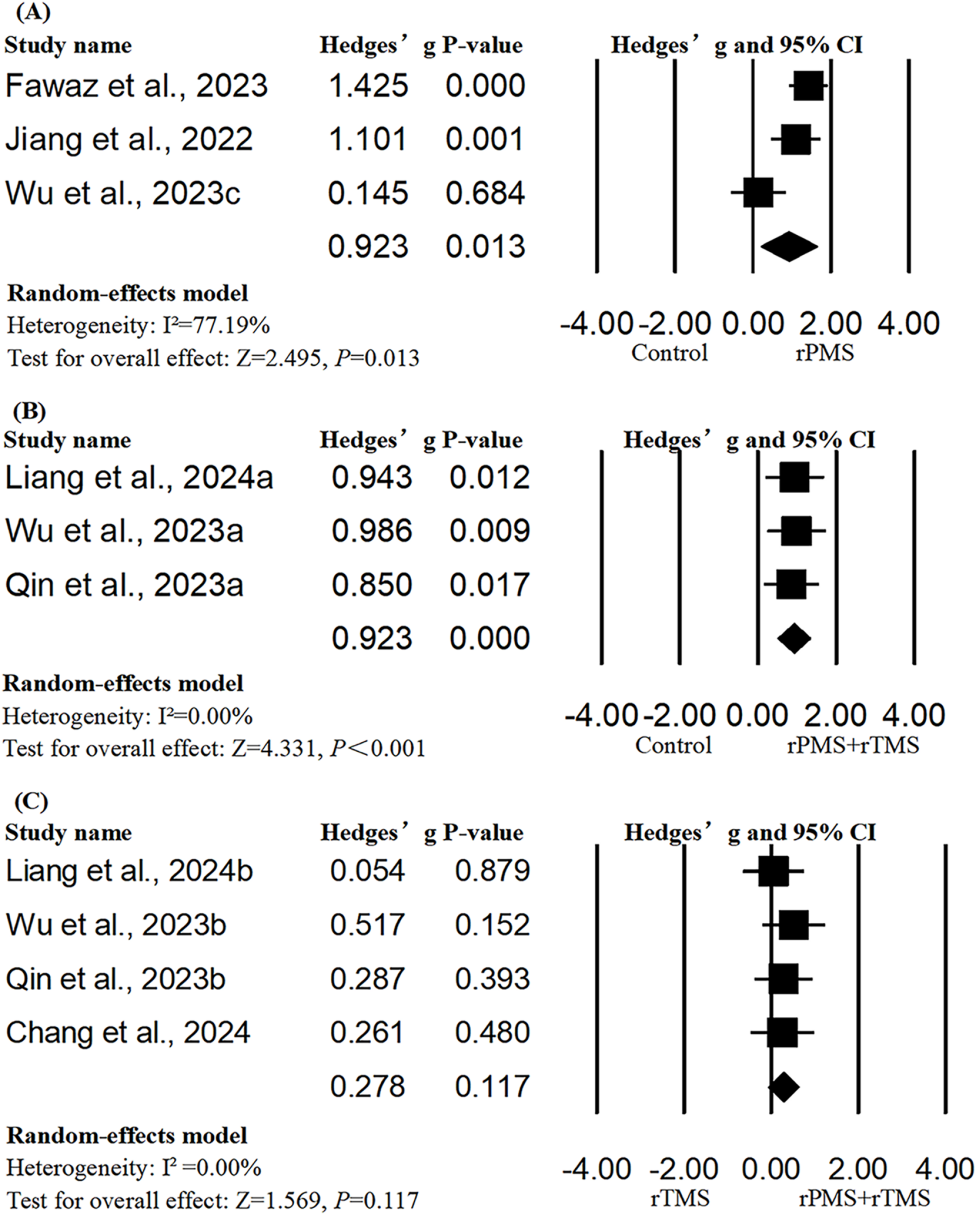
Forest plots of the pooled outcomes. Activities of Daily Living (ADL): Group **A**: Hedges’ g = 0.923, *p* = 0.013, I²=77.19; Egger’s test: *p* = 0.29; Group **B**: Hedges’ g = 0.923, *p* < 0.001, I²=0; Egger’s test: *p* = 0.15; Group **C**: Hedges’ g = 0.278, *p* = 0.117, I²=0; Egger’s test: *p* = 0.88)

A total of six studies with 10 units of analysis were included in the meta-analysis of ADL [12, 32–34, 36, 38]. The results of meta-analysis showed that both rPMS alone and rPMS in combination with rTMS showed more significant benefits than the control group in improving the activity levels of the participant (rPMS: Hedges’ g = 0.923, *p* = 0.013; rPMS + rTMS: Hedges’ g = 0.923, *p* < 0.001; Fig. 3), and the significant results were robust to leave-one-out sensitivity analysis (rPMS: Hedges’ g from 0.198 to 1.647; rPMS + rTMS: Hedges’ g from 0.505 to 1.34). However, rPMS combined with rTMS was not more effective than the use of rTMS alone (Hedges’ g = 0.278, *p* = 0.117). There was no evidence of publication bias in meta-analyses of all subgroups (Figures S4–S6).

Univariate meta-regression revealed that total pulses (*p* = 0.003), number of pulses per session (*p* < 0.001), total pulses per site (*p* = 0.005) and number of pulses per site per session (*p* < 0.001) were significant predictors regarding the benefits from rPMS intervention on ADL (Table S5). However, from the scatter plots, the significant findings seemed to be driven by the study by Fawaz et al. After removing this study, number of pulses per session (*p* = 0.029) remained a significant predictor of effect sizes of ADL (Figure S7).

### Upper limb muscle spasticity

Due to insufficient data (< 3 studies in each subgroup), we were not able to conduct a quantitative analysis of muscle spasticity. Among six controlled studies, four revealed positive effects on relieving spasticity [12, 39, 43, 45], including one study combining rPMS and rTMS [12] and three studies using rPMS alone [39, 43, 45], while two studies reported no significant effects following treatment with rPMS alone [38, 47]. Additionally, two single-group studies, one of which used rPMS alone [46] and the other of which applied combination intervention [35], reported positive effects regarding reduced spasticity.

### Neuromodulatory effects

A total of three studies evaluated changes in neuroimaging or neurophysiological outcomes, using electroencephalography (EEG) [43], functional magnetic resonance imaging (fMRI) [12], and TMS-based outcomes [32]. Qin et al. demonstrated that the cortical sensorimotor area and cerebellum were activated following the combined use of low frequency rTMS and rPMS, compared to sham stimulation. Furthermore, Liang et al. reported a significant decrease of short interval intracortical inhibition (SICI) in the contralesional hemisphere induced by rPMS associated with high frequency rTMS. Similarly, event-related desynchronization (ERD, an index of cortical activation) in contralesional hemisphere was decreased after a single rPMS session [43]. In summary, rPMS alone or in combination with rTMS seems to be able to modulate the bilateral hemispheric activities in poststroke brains.

## Discussion

The main results of meta-analysis revealed that (1) rPMS alone and rPMS combined with rTMS both significantly improved upper limb motor function recovery and activities independence in poststroke patients, as compared to the control; (2) the number of stimulation pulses per session and total pulses were positively correlated with the effect size of rPMS on ADL, indicating that the effect of rPMS may demonstrate a dose-dependent outcome; and (3) no statistical evidence was found to support the hypothesis that a combined use of rPMS and rTMS is more effective than rTMS alone on improving motor function and ADL.

Despite this, the parameters and timing of the rPMS may affect the treatment effect—for example, frequency, doses, targets, and time since stroke could all play roles in the treatment’s effectiveness [48, 49]. In the present review, the dose of rPMS pulses appeared to have a significant impact on the effects in activities participation. Behavioral changes could result from improved neuroplasticity induced by interventions in stroke rehabilitation [50]. Similarly, Gallasch et al. also revealed that a total number of 15,000 single pulses of rPMS drives sensorimotor cortical excitability over the contralateral M1 and S1, but this effect was not observed after delivering a low dose of 6000 stimulation pulses [51]. It may be because the effect of rPMS on ADL was dose-dependent and that therefore the insufficient dose was not strong enough to elicit cortical plasticity changes. This was parallel with the dose-dependent effect of rTMS reported by previous studies. A higher dose of rTMS was also associated with a higher level of cortical excitability and a greater increase in clinical effectiveness in poststroke motor rehabilitation than lower dose protocols [52–54]. Therefore, non-invasive neuromodulation therapy, applied over either the peripheral or central nervous system, appears to elicit a dose-dependent response in poststroke rehabilitation. Additionally, we observed numerically larger effect sizes in the two studies involving acute stroke patients (within one month after stroke onset) [38, 44]. However, our regression analysis using the mean months after stroke did not reveal an impact of chronicity on recovery outcomes. This may be due to the fact that many of the included studies featured a mixed stage of stroke patients, which may have diluted the effects associated with chronicity and obscured potential trends in recovery outcomes.

However, contrary to our expectation, after pooling the sample sizes of multiple experiments, we found that there were no stronger synergistic effects when rPMS was combined with rTMS, compared to using rTMS alone. Firstly, in these included studies the protocols of rPMS combined with rTMS varied. Most of studies delivered the rPMS and rTMS one after the other, rather than using paired associative stimulation [12, 33, 34], i.e., a paired, central-associated, peripheral stimulation involves delivering a single pulse of rTMS to the primary motor cortex (M1) and a single pulse of rPMS to the afferent fibers up to the primary somatosensory cortex (S1), alternately. In contrast to the effect of separate delivery of the two stimulations, the timing-dependent effect of the stimulation relative to the afferent input may be very different. Previous studies have revealed that the form of paired associative stimulation may enhance its efficacy on modulating M1 excitability in healthy subjects [55, 56]. Also, PAS had also been reported as effective in improving motor learning and accelerating motor recovery in patients with stroke by inducing associative neuroplasticity and reducing intracortical inhibition [32, 57, 58]. Secondly, the non-inferiority of the combined treatment may be because these two treatments may have similar effect mechanisms on improving motor performance by mediating similar cortex region plasticity and reducing intracortical inhibition [12, 51, 57, 59]. Previous studies have indicated that rTMS was very effective in facilitating motor relearning and recovery as well as ADL in stroke rehabilitation by modulating M1 excitability [60–62]. Therefore, the synergistic effects of combining the two are not additive. The effect of rTMS on cortical plasticity may have already reached a ceiling effect, so that combining it with rPMS cannot further increase its clinical effectiveness. Overall, whether rPMS was used alone or in combination with rTMS, the optimal matching protocols, regarding targets, dose, frequency, intensity and duration, timing, and lasting effects need to be explored further.

### Limitations

This review was not free from limitations. Firstly, due to heterogeneous quality of the rPMS intensity used in different studies, we performed a qualitative analysis. Because most of them applied supra-threshold stimulation, subgroup analysis was not performed. Secondly, although we analyzed the potential relationship between stimulation parameters and clinical outcomes, the current review was unable to identify clinical cutoff values for effective stimulation doses and the number of treatment sessions due to the limited number of included studies. Thirdly, substantial heterogeneity and publication bias was identified in the rPMS-only intervention subgroup, probably due to the small sample sizes, the clinical characteristics of participants, and inconsistent rPMS protocols and methodologies among the studies. We hope that future large-scale studies will be more transparent in their reporting.

## Conclusions

rPMS alone or in combination with rTMS can effectively promote upper-extremity motor functional recovery and activity independence in poststroke patients, indicating that both bottom-up and top-down approaches are equally useful. Simple combination use may not necessarily produce better therapeutic effects than using rTMS alone, although developing rPMS protocols with higher doses may generate better responsiveness. Although rPMS is able to modulate the excitability and intracortical inhibitory activities of both hemispheres, its specific underlying mechanism remains largely unclear, awaiting further investigation.

## Electronic supplementary material

Below is the link to the electronic supplementary material.

Supplementary Material 1: Table S1: Search strategy keywords. Table S2: Methodological quality assessment of included studies with parallel design. Table S3: Characteristics of stimulation protocols of studies investigating the effects of rPMS combined with rTMS. Table S4: Results of meta-regression of moderators for the effect sizes of rPMS intervention in FMA-UE scores. Table S5: Results of meta-regression of moderators for the effect sizes of rPMS intervention in ADL. Figs. S1–S6: The funnel plots for the meta-analysis regarding the effects of rPMS alone or in combination with rTMS on various outcomes. Fig. S7: Meta-regression lines of the association between the effect size of ADL and significant moderators.

## Data Availability

The data that support the findings of this study are available from the corresponding author, upon request.

## Abbreviations

ADL: Activities of daily living
FMA-UE: Fugl-Meyer Assessment-Upper Extremity
FIM: Functional independence measure
M1: Primary motor cortex
MBI: Modified Barthel Index
PAS: Paired associative stimulation
rPMS: Repetitive peripheral magnetic stimulation
rTMS: Repetitive transcranial magnetic stimulation
S1: Primary somatosensory cortex
