# Supplementary material for: Repetitive peripheral magnetic stimulation alone or in combination with repetitive transcranial magnetic stimulation in poststroke rehabilitation: a systematic review and meta-analysis
Source: J Neuroeng Rehabil. 2024 Oct 16;21:181. doi: 10.1186/s12984-024-01486-8 (PMC11481378; doi:10.1186/s12984-024-01486-8)
Supplement: Supplementary file 1 — Supplementary Material 1: Table S1: Search strategy keywords. Table S2: Methodological quality assessment of included studies with parallel design. Table S3: Characteristics of stimulation protocols of studies investigating the effects of rPMS combined with rTMS. Table S4: Results of meta-regression of moderators for the effect sizes of rPMS intervention in FMA-UE scores. Table S5: Results of meta-regression of moderators for the effect sizes of rPMS intervention in ADL. Figs. S1–S6: The funnel plots for the meta-analysis regarding the effects of rPMS alone or in combination with rTMS on various outcomes. Fig. S7: Meta-regression lines of the association between the effect size of ADL and significant moderators. [file 12984_2024_1486_MOESM1_ESM.docx]

**Repetitive peripheral magnetic stimulation alone or in combination with repetitive transcranial magnetic stimulation in poststroke rehabilitation: a systematic review and meta-analysis**

**Table S1. Search Strategy Keywords**

| **Main term** | **Keywords** |
| --- | --- |
| 1. Stroke | “hemiplegia or stroke or cerebrovascular accident or cerebral stroke or cerebral hemorrhage or cerebral infarction or cerebral ischemia or poststroke or paresis or paretic” |
| 2. Upper limb | “upper limb or upper limbs or hand or wrist or finger or elbow or shoulder or upper extremity or upper extremities” |
| 3. Peripheral magnetic stimulation | “(peripheral or nerve or muscle or spine or spinal) AND (magnetic or electromagnetic or electro-magnetic or transcranial magnetic stimulation)” |
| **Final search items** | 1 AND 2 AND 3 |

**Table S2. Methodological quality assessment of included studies with parallel design**

| **Study** | **Item 1** | **Item 2** | **Item 3** | **Item 4** | **Item 5** | **Item 6** | **Item 7** | **Item 8** | **Item 9** | **Item 10** | **Total scores** |
| --- | --- | --- | --- | --- | --- | --- | --- | --- | --- | --- | --- |
| Krewer et al., 2014 | 1 | 1 | 1 | 1 | 0 | 1 | 1 | 1 | 1 | 1 | 9 |
| Yang et al., 2018 | 0 | 0 | 1 | 0 | 0 | 0 | 1 | 1 | 1 | 1 | 5 |
| Obayashi et al., 2020 | 1 | 0 | 1 | 0 | 0 | 0 | 1 | 1 | 1 | 1 | 6 |
| Chen S et al., 2020 | 0 | 0 | 1 | 1 | 1 | 1 | 1 | 1 | 1 | 1 | 8 |
| Ke et al., 2022 | 1 | 0 | 1 | 1 | 0 | 1 | 1 | 1 | 1 | 1 | 8 |
| Jiang et al., 2022 | 1 | 1 | 1 | 1 | 1 | 1 | 1 | 1 | 1 | 1 | 10 |
| Qin et al., 2023 | 1 | 0 | 1 | 1 | 0 | 1 | 0 | 1 | 1 | 1 | 7 |
| Wu et al., 2023 | 1 | 1 | 1 | 1 | 0 | 1 | 1 | 1 | 1 | 1 | 9 |
| Fawaz et al., 2023 | 1 | 1 | 1 | 1 | 0 | 1 | 1 | 1 | 1 | 1 | 9 |
| Liang et al., 2024 | 1 | 0 | 1 | 1 | 0 | 1 | 1 | 1 | 1 | 1 | 8 |
| Change et al., 2024 | 1 | 1 | 1 | 1 | 1 | 1 | 1 | 1 | 1 | 1 | 10 |
| Fujimura et al., 2024 | 1 | 1 | 1 | 0 | 0 | 1 | 1 | 1 | 1 | 1 | 8 |

Notes: The Physiotherapy Evidence Database (PEDro) scale consists of 10 items: item 1: random allocation; item 2: concealment of allocation; item 3: baseline equivalence; item 4: blinding procedure (subjects); item 5: blinding procedure (therapists); item 6: blinding procedure (assessors); item 7: adequate follow-up; item 8: intention to treat analysis; item 9: between-group statistical analysis; item 10: measurement of data variability and point estimates.

**Table S3. Characteristics of stimulation protocols of studies investigating the effects of rPMS combined with rTMS**

| **Study** | **TMS protocols** | | | **PMS protocols** | | | |
| --- | --- | --- | --- | --- | --- | --- | --- |
| **Author** | **Dose (pulses)** | **Intensity & frequency** | **Stimulation target** | **Dose & type of coil** | **Intensity & frequency** | **Duration** | **Stimulation target** |
| Qin et al., 2023 | rTMS-1200 | 1Hz  90% RMT | contralesional M1 | rPMS-1200  Figure-of-eight coil  Delivered immediately after LF-rTMS | 10Hz  Minimum intensity inducing subtle visible muscle contractions | Once a day, 5 days/week, 8 weeks; 40 sessions | Erb’s point of the paretic upper limb |
| Wu et al., 2023 | rTMS-1000 | 10Hz  80% RMT | ipsilesional M1 | rPMS-1000  Circular coil  Delivered after rTMS | 10Hz  The lowest stimulation intensity that can trigger muscle contraction | Once a day, 5 days/week; 3 weeks  15 sessions | C5-T1 nerve root over the paretic side |
| Yang et al., 2023 | rTMS-780 | 10Hz  80% RMT | contralesional M1 | rPMS-750, 5Hz (MAS≥1+)  rPMS-5100, 20 Hz (MAS＜1+)  Figure-of-eight  Performing separately from rTMS | 5Hz and 20Hz  100% RMT | Once a day, 15 days, 15 sessions | Flexor and extensor of the paretic elbow and wrist |
| Liang et al., 2024 | rTMS-1200 | 5Hz  80% AMT | ipsilesional M1 | rPMS-1200  Double-ended circular coil  Delivered 20 ms after rTMS (paired) | 5Hz  80% RMT | Once a day, 5 days/week, 4 weeks,  20 sessions | The C7 nerve root over the paretic side |
| Chang et al., 2024 | iTBS-600 | 50Hz  70% of RMT | ipsilesional M1 | iTBS-600  Figure-of-eight coil  Delivered 10 minutes before central iTBS | 5Hz  Intensity inducing muscle contraction of extensor carpi radialis muscle | Once a day, 5 days/week, 2 weeks  10 sessions | Radial nerve of the paretic upper limb (radial groove) |

Abbreviations: AMT: Active motor threshold; RMT: Resting motor threshold; M1: Primary motor cortex; iTBS: Intermittent theta burst stimulation; MAS: Modified Ashworth scale.

**Table S4. Results of meta-regression of moderators for the effect sizes of rPMS intervention in FMA-UE scores**

| **Moderators** | N | Univariate coefficient | Z-Value | *p*-Value |
| --- | --- | --- | --- | --- |
| ***rPMS parameters:*** |  |  |  |  |
| Number of rPMS sessions | 11 | -0.0050 | -0.29 | 0.772 |
| Number of pulses per site per session | 10 | -0.0001 | -0.89 | 0.373 |
| Total pulses per site | 10 | <-0.00001 | -0.71 | 0.479 |
| Total pulses | 11 | <-0.00001 | -0.73 | 0.418 |
| Number of pulses per session | 11 | <0.00001 | -0.78 | 0.434 |
| Frequency | 12 | -0.0186 | -1.04 | 0.297 |
| ***Demographics:*** |  |  |  |  |
| Mean age (years) | 13 | 0.0377 | 0.96 | 0.339 |
| Percentage of males | 13 | 0.0016 | 0.07 | 0.945 |
| ***Clinical profiles:*** |  |  |  |  |
| Mean time since stroke (months) | 12 | -0.0294 | -1.07 | 0.283 |
| Mean baseline severity (FMA-UE) | 13 | -0.0102 | -0.39 | 0.697 |
| Percentage of ischemic strokes | 9 | 0.0180 | 1.38 | 0.168 |

**p*<0.05***p*<0.01

**Table S5. Results of meta-regression of moderators for the effect sizes of rPMS intervention in ADL**

| **Moderators** | N | Univariate coefficient | Z-Value | *p*-Value |
| --- | --- | --- | --- | --- |
| ***rPMS parameters:*** |  |  |  |  |
| Number of rPMS sessions | 7 | -0.0147 | -0.58 | 0.559 |
| Number of pulses per site per session | 6 | 0.0003 | 3.93 | **<0.001**** |
| Total pulses per site | 6 | <0.00001 | 2.79 | **0.005**** |
| Total pulses | 7 | <0.00001 | 3.02 | **0.003**** |
| Number of pulses per session | 7 | 0.0001 | 3.36 | **<0.001**** |
| Frequency | 7 | 0.0110 | 0.72 | 0.473 |
| ***Demographics:*** |  |  |  |  |
| Mean age (years) | 7 | 0.0163 | 0.60 | 0.548 |
| Percentage of males | 7 | -0.0051 | -0.21 | 0.830 |
| ***Clinical profiles:*** |  |  |  |  |
| Mean time since stroke (months) | 6 | 0.1276 | 1.03 | 0.303 |
| Mean baseline severity (FMA-UE) | 7 | 0.0133 | 0.44 | 0.663 |
| Mean baseline of ADL | 7 | -0.0151 | -0.84 | 0.402 |
| Percentage of ischemic strokes | 4 | -0.0085 | -0.19 | 0.853 |

**p*<0.05***p*<0.01

**Figure S1.** The funnel plot for the meta-analysis regarding the outcome of FMA-UE after intervention with rPMS alone. The funnel plot for Figure S2 shows no evidence of publication bias.

**Figure S2.** The funnel plot for the meta-analysis regarding the outcome of FMA-UE after intervention with both rPMS and rTMS. The funnel plot for Figure S3 shows no evidence of publication bias.

**Figure S3.** The funnel plot for the meta-analysis regarding the outcome of FMA-UE after intervention with both rPMS and rTMS, in contrast to intervention with rTMS alone. The funnel plot for Figure S4 shows no evidence of publication bias.


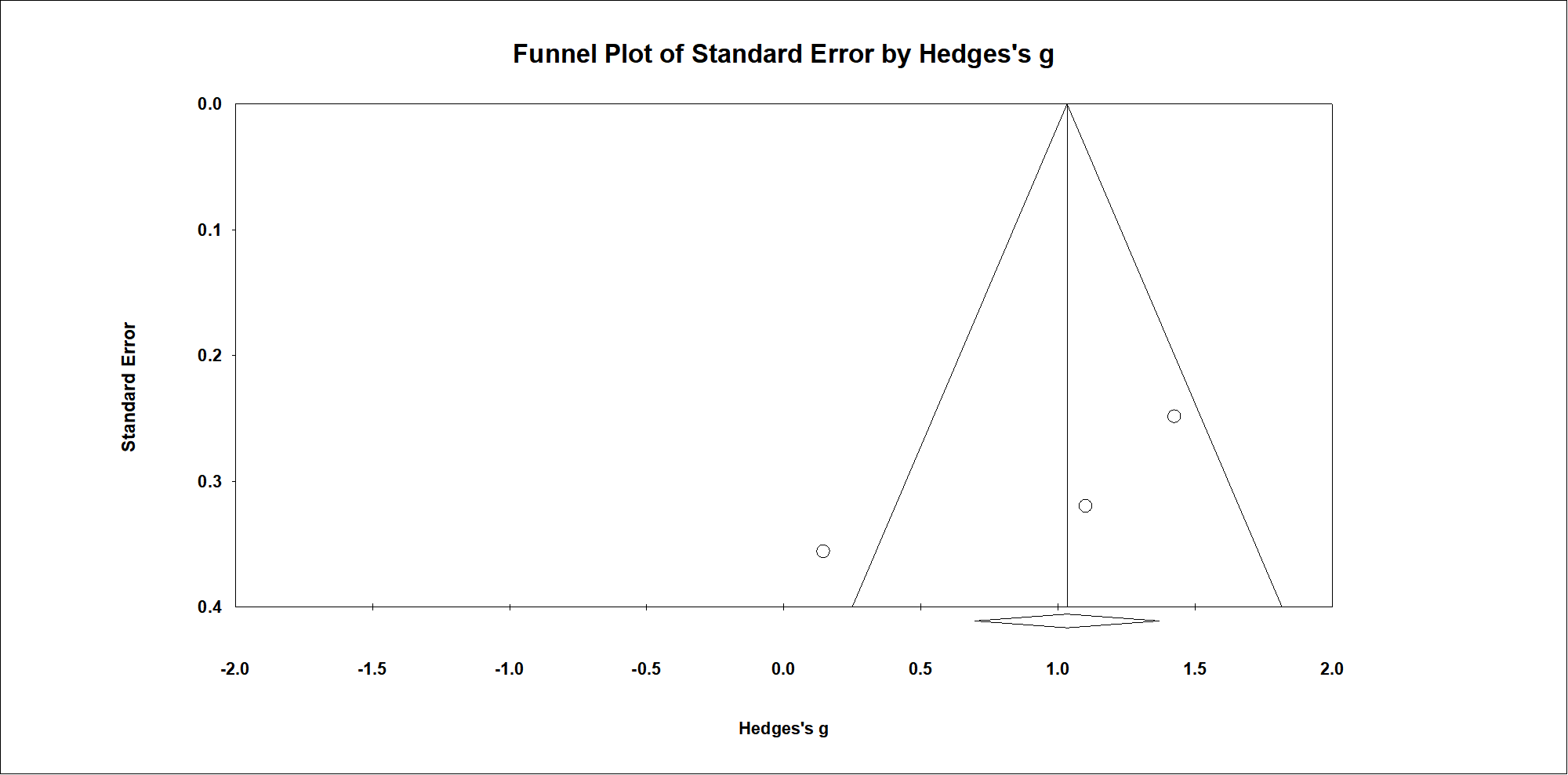


**Figure S4.** The funnel plot for the meta-analysis regarding the outcome of ADL after intervention with rPMS alone. The funnel plot for Figure S5 shows no evidence of publication bias.

**Figure S5.** The funnel plot for the meta-analysis regarding the outcome of ADL after intervention with both rPMS and rTMS. The funnel plot for Figure S6 shows no evidence of publication bias.

**Figure S6.** The funnel plot for the meta-analysis regarding the outcome of ADL after intervention with both rPMS and rTMS, in contrast to intervention with rTMS alone. The funnel plot for Figure S7 shows no evidence of publication bias.


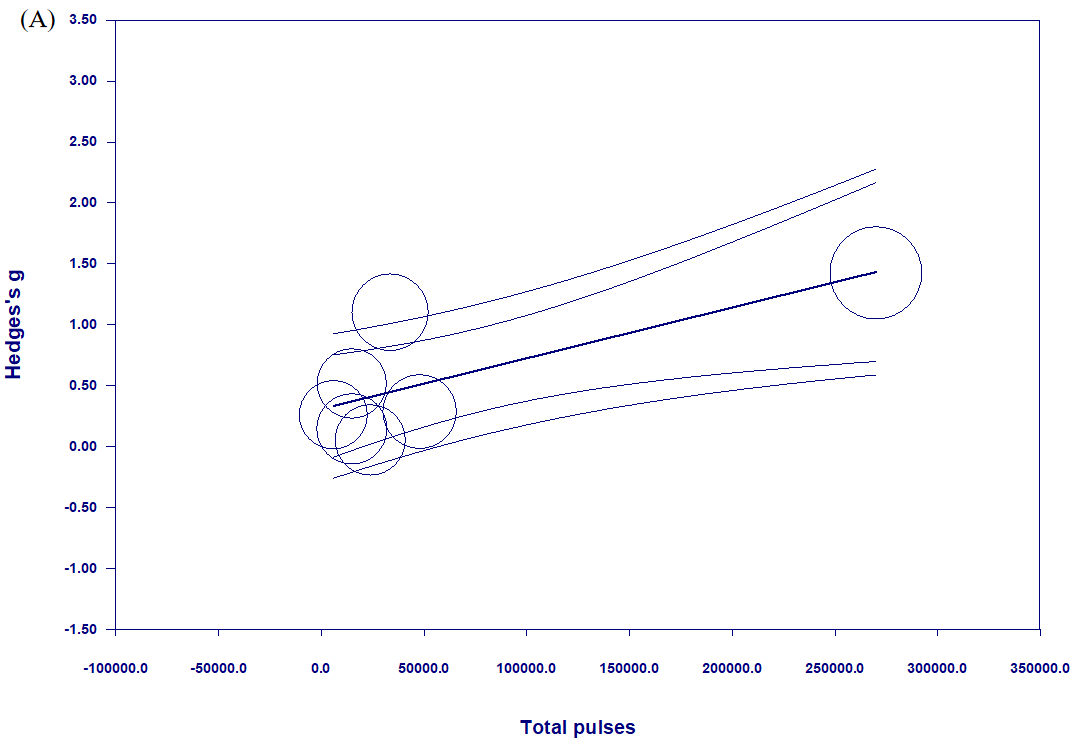

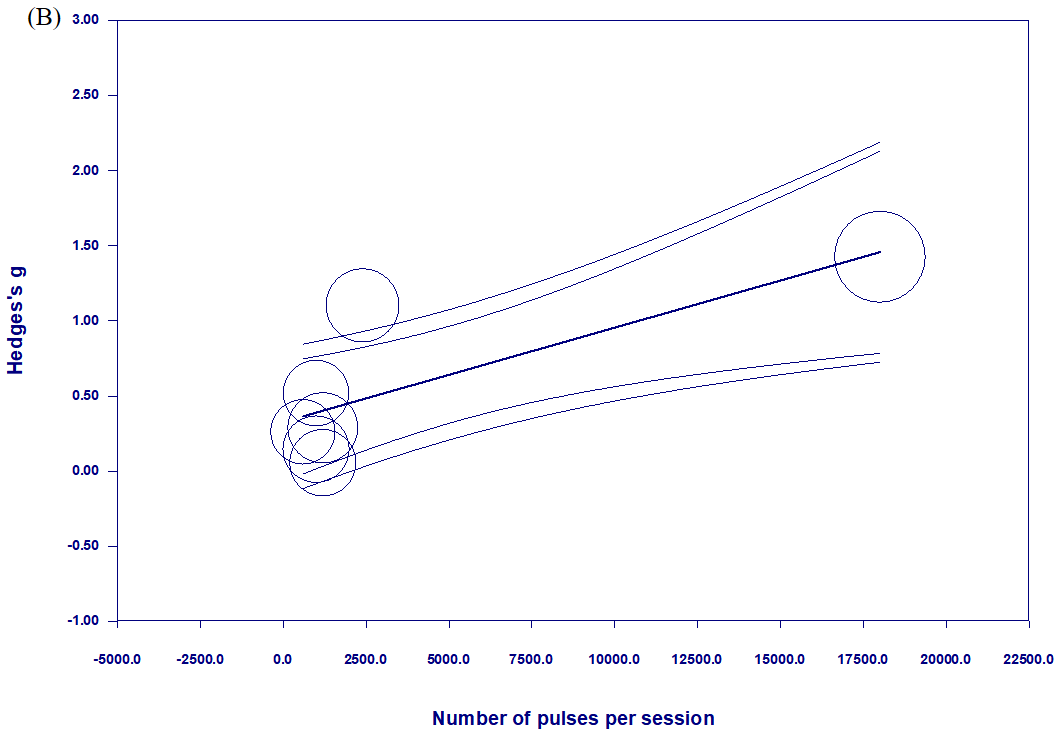


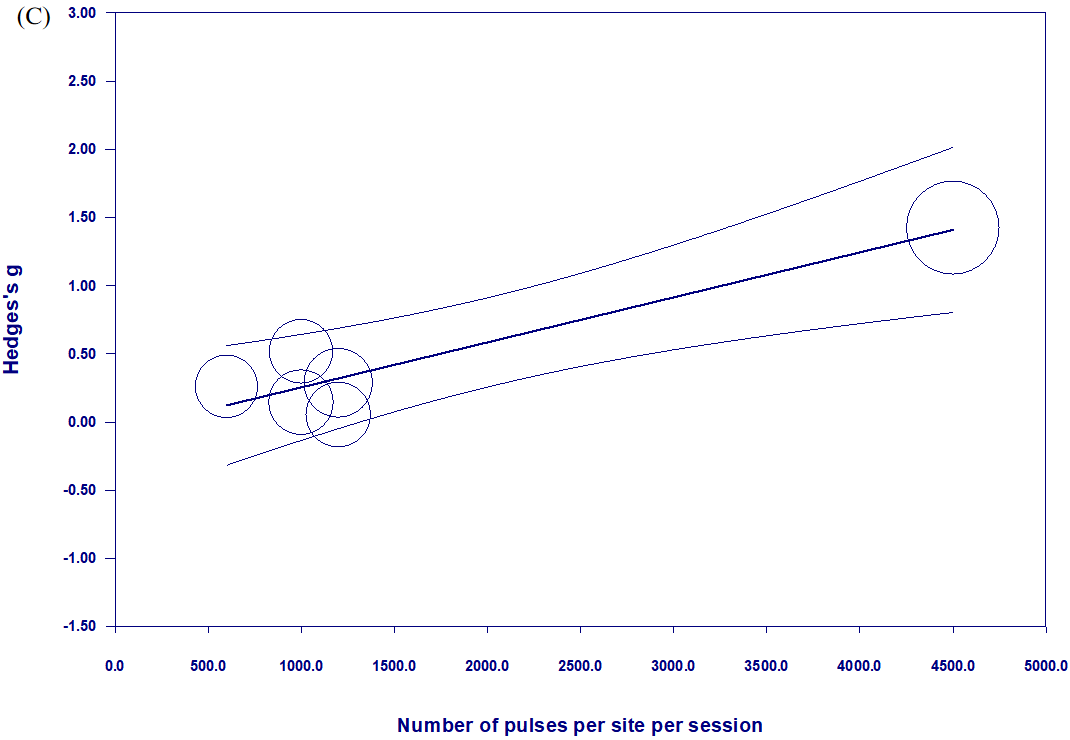

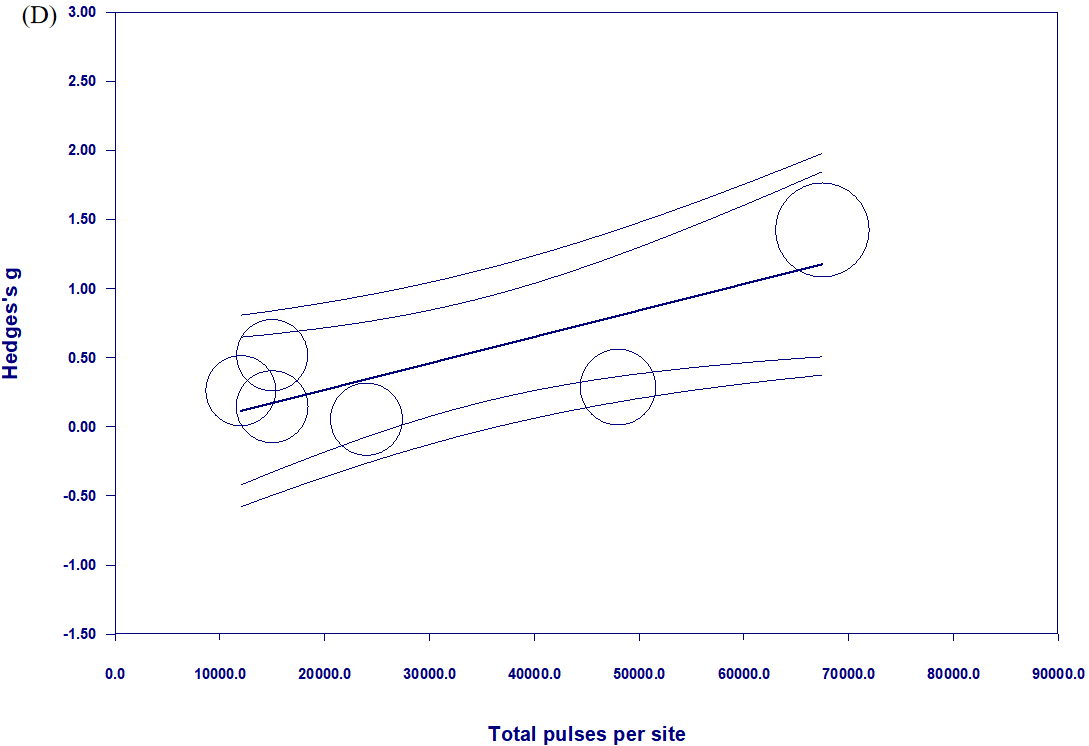


**Figure S7.** Meta-regression of the association between the effect size of ADL and: (A) total pulses; (B) number of pulses per session; (C) number of pulses per site per session; and (D) total pulses per site. Each study was represented by a circle proportional to its weight in the analysis.
